# Anatomical Researches on the Excretory Ducts of the Lachrymal Gland

**Published:** 1844-04-06

**Authors:** 


### ANATOMICAL RESEARCHES ON THE EXCRETORY DUCTS OF THE LACHRYMAL GLAND.

M. Gosselin, prosector to the Faculty of Medicine, Paris, has recently been making some inquiries with a view to determine the exact number of the excretory ducts of the lachrymal gland, respecting which anatomists do not agree. Stenon, who first discovered them, in the calf, says there are thirteen or fourteen, but only represents eleven, in his plates of the eye. Most anatomical writers since Stenon, have stated that they are ten or twelve in number. Monro, however, who first saw them in the human eye, was only able to inject two.

M. Gosselin began by looking for the ducts in the sheep, in which animal they were discovered by Stenon. He found the orifices of two ducts, with ease, but although he examined the mucous surface with the greatest attention, he could never discover any other orifices. On introducing a small lymphatic injection tube into the two foramina, he found that the mercury penetrated into every region of the lachrymal gland, without any other ducts becoming visible; and therefore concluded that the two he had injected were, in reality, the only ones which existed. As the same experiment, performed on the eye of another sheep, was attended with the same result, M. Gosselin thinks that, generally speaking, there are only two excretory canals to the lachrymal duct of the sheep, although more are said to exist by Stenon.

The lachrymal gland of an ox was next examined. In this animal the ducts are large, and the foramina easily perceived, opening obliquely under the conjunctiva, in the same manner as the ureters in the bladder. Only five were discovered, and it was not found possible to inject them, owing probably to the density of the glandular tissue of the organ in this animal.

M. Gosselin then turned his attention to the human eye, but found great difficulty in prosecuting his researches. The orifices of the ducts are so minute that it is scarcely possible to perceive them at first. When the mucous membrane is macerated, as anatomists direct, in coloured solutions, there are so many points coloured that it is impossible to look upon them all as foramina. By means of the minute glass tubes for lymphatic injections, after many attempts, M. Gosselin, succeeded however, in injecting the ducts and the lachrymal gland, but never found more than two ducts going directly to the gland, so as to render possible its injection by their means. The other ducts, six or eight in number, led to the small glandulæ, or prolongations of the orbital gland, which are known to anatomists under the name of "the palpebral portions of the lachrymal gland."

The plan he adopted to make these injections is as follows: The lachrymal gland was separated from the orbit, along with a certain portion of the superior palpebra. The eyelid being then spread over the indicator of the left hand, the mucous membrane was firmly stretched. A certain number of small points, situated on an oblique line, downwards and outwards, were then seen, below the reflection of the conjunctiva. The smallest glass tube that could be made was introduced with the greatest precaution, successively, into each of these foramina, and the mercury was allowed to flow during ten or fifteen minutes.

These researches have a practical result, says M. Gosselin. After several operations in which the lachrymal gland had been extirpated, it was remarked with surprise that the eye was still lubricated with the lachrymal fluid, and retained its humidity and polish. This circumstance was owing, no doubt, to the integrity of the palpebral glandulæ, which having separate excretory ducts, continued to supply the eye with a sufficient quantity of the lachrymal fluid to perfectly lubricate its surface. It is therefore evident that whenever it is necessary to extirpate the lachrymal gland, there need be no fear entertained with regard to the integrity of the functions of the eye, if the glandulæ of the superior palpebra remain intact.—*London Lancet, Jan.* 27, 1844.

